# Supplementary material for: Superelastic NiTi Functional Components by High-Precision Laser Powder Bed Fusion Process: The Critical Roles of Energy Density and Minimal Feature Size
Source: Micromachines (Basel). 2023 Jul 18;14(7):1436. doi: 10.3390/mi14071436 (PMC10383407; doi:10.3390/mi14071436)
Supplement: Supplementary file 1 [file micromachines-14-01436-s001.zip › micromachines-2472335 Figures.pdf]

micromachines

MDPI

Supplementary material

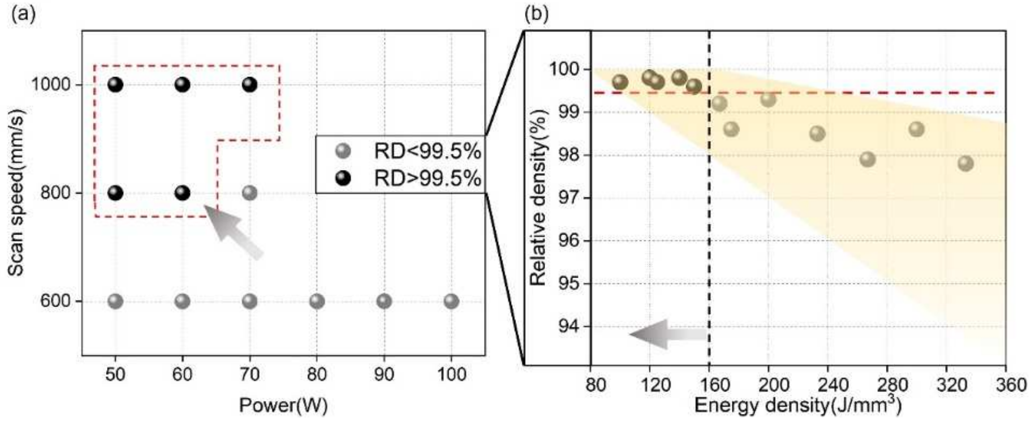

Figure. S1 RDs of as-printed cubes

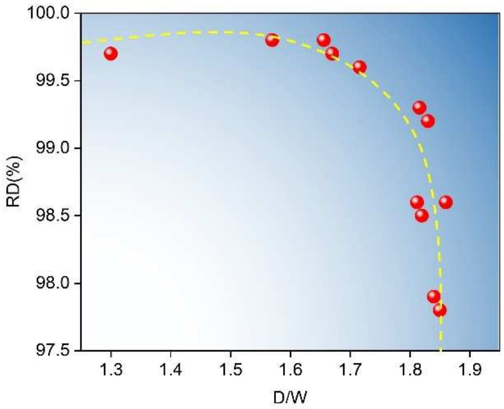

Figure. S2 The relationship between D/W and RD of NiTi samples

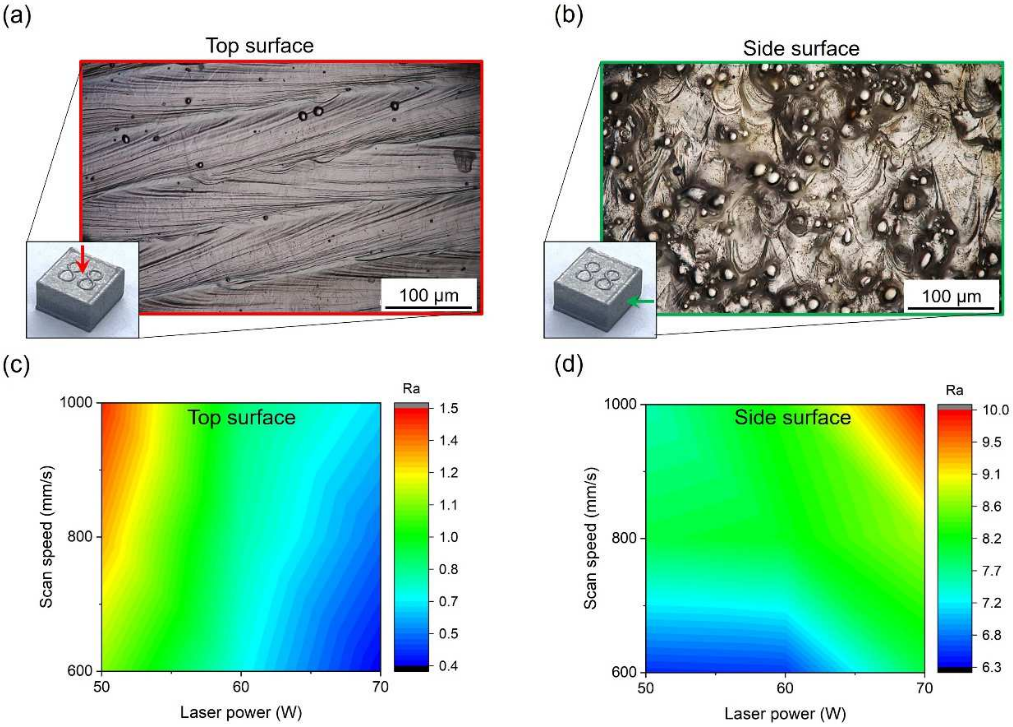

Figure. S3 Surface roughness characterization of cubes fabricated using different parameters: (a) top surface morphology, (b) side surface morphology, (c) Ra of top surfaces obtained using different parameters, (d) Ra of side surfaces obtained using different parameters

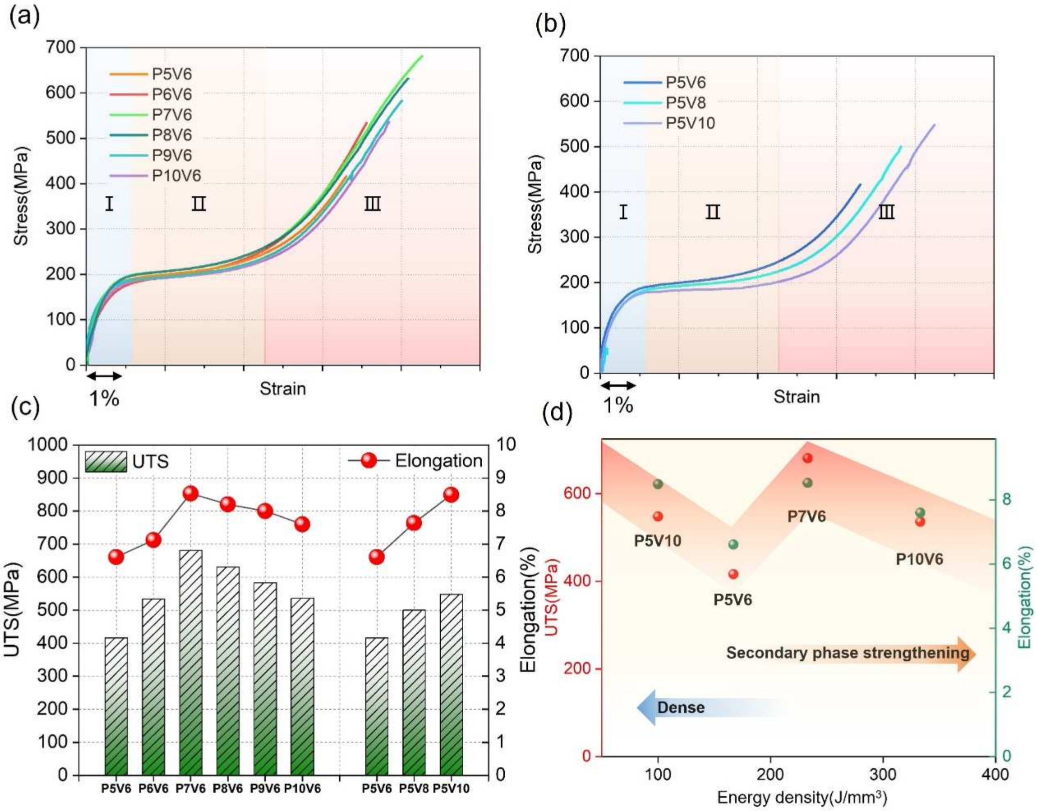

Figure. S4 Representative tensile curves of samples fabricated with varied (a) laser power and (b) scan speed, (c) UTS and elongation values of NiTi samples with different parameters, (d) UTS and elongation values of four typical samples versus energy density

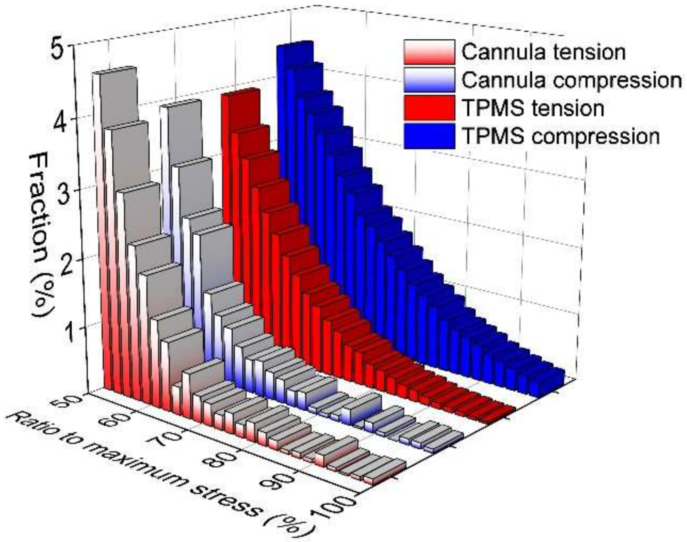

Figure. S5 Tension and compression stress conditions of elements extracted from FE simulation of the G-TPMS and cannula tip
